# Participatory science in urban soil research: A framework for overcoming challenges and expanding public engagement

**DOI:** 10.1016/j.isci.2025.112361

**Published:** 2025-04-06

**Authors:** Anna A. Paltseva

**Affiliations:** 1 Departments of Agronomy and Horticulture and Landscape Architecture, Purdue University, West Lafayette, IN 47907, USA

**Keywords:** Earth sciences, Soil science, Social sciences, Education, Research methodology social sciences

## Abstract

Urban soils play a crucial role in supporting city sustainability, including green infrastructure, food production, water regulation, and climate resilience. Yet their complexity, compounded by environmental risks, pose challenges for research and policy. This article presents a participatory science framework that engages communities in soil monitoring and management through co-designed projects, educational outreach, and simplified data collection protocols. By integrating digital tools and real-time mapping, the framework enhances data accuracy while fostering environmental stewardship. Grounded in interdisciplinary collaboration, the framework connects scientific research, technological support, community engagement, and policy advocacy to improve urban soil health. Demonstrated through case studies and practical strategies, this approach builds capacity for sustained community involvement and equitable environmental decision-making. The broader significance lies in the framework’s ability to foster reciprocal relationships between scientists and communities, where shared knowledge, action, and responsibility strengthen urban soil stewardship.

## Graphical abstract

**Figure:**
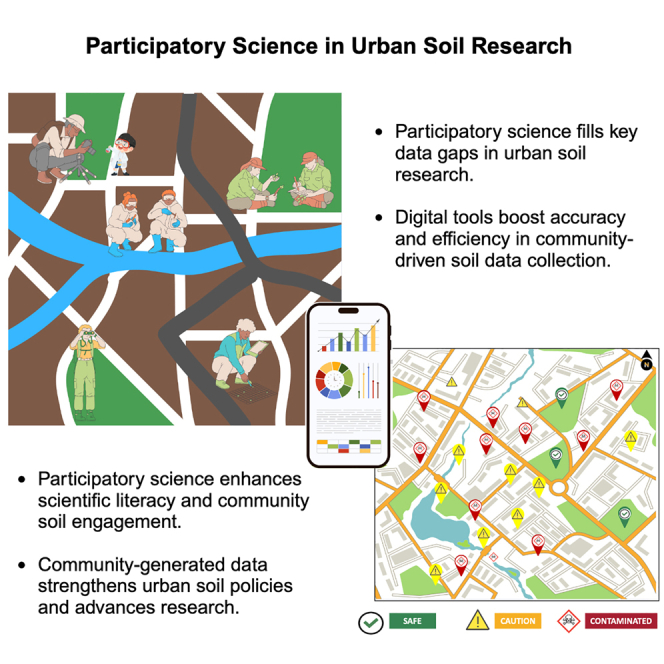

## Introduction

### Urban soils: Underappreciated pillars of metropolitan sustainability

Urban soils are an invaluable resource that underpins biodiversity, water regulation, and climate resilience in rapidly growing cities—supporting both environmental quality and public well-being.^1^^,^^2^^,^^3^ Urban soils are essential for sustaining green spaces, enabling urban agriculture, and facilitating carbon sequestration—collectively improving air quality, enhancing food security, and aiding climate change mitigation.^4^^,^^5^^,^^6^^,^^7^^,^^8^ The proper management of urban soils also protects public health by mitigating contamination from harmful elements such as heavy metals.^5^^,^^9^^,^^10^

With the proportion of the global population living in cities expected to rise from 55% to 80% by 2050, urban soils present both opportunities and challenges.^11^ While they provide a platform for public education on soil health and environmental topics, their accessibility raises concerns about human exposure to contaminants, soil degradation, compaction, erosion, loss of biodiversity, and disrupted ecosystem services.^6^ As cities expand, the need for precise, informed management of urban soils becomes increasingly urgent. Barriers such as data gaps, limited public awareness of soil functions, and logistical difficulties in accessing densely developed areas continue to obstruct progress in urban soil science.^12^ Urban soils exhibit significant heterogeneity due to the diverse land uses and disturbance histories inherent in urban landscapes. These variations, even over short distances, complicate data collection and analysis, often rendering traditional soil surveys inadequate for capturing the true complexity of urban soil systems.^13^^,^^14^

Public awareness is another pressing issue. Soils are commonly perceived as mere substrates for plant growth rather than as vital resources that require active management and conservation. Unlike air or water, the complexity and less visible nature contribute to its undervaluation in environmental discourse. Additionally, urban settings present unique challenges for comprehensive soil surveys.^15^ Cities are characterized by impervious surfaces and restricted access, which impede data collection efforts. In many cases, contamination - particularly from heavy metals - requires specialized testing and remediation that further complicate urban soil management.^12^^,^^16^ Addressing these challenges requires innovative approaches.

### The role of participatory science in urban soil research

Participatory science, which involves the public in scientific research through collaboration with professional scientists and institutions to formulate research questions, collect data, and interpret results,^17^ is a promising solution. Also known as citizen science, community science, or public participation in scientific research,^17^ it has been widely used in recent literature to describe community-driven initiatives. However, recognizing the growing emphasis on inclusivity, the term “participatory science” is adopted throughout this article to reflect the engagement of all individuals, regardless of citizenship status, in environmental research. For consistency with the literature, “citizen science” is retained in the citations.

Participatory science projects often involve collaboration between scientists and non-scientists, with the goal of gathering, sharing, and studying original data. These initiatives can enhance participants’ scientific literacy while producing valuable field observations that monitor a range of environmental conditions.^18^^,^^19^ The rise of technological advancements, such as smartphones with mapping applications and GPS capabilities, has further facilitated citizen science.^20^ Interactive websites enabling citizens to input and edit their observations have democratized environmental data collection, making it more accessible.^21^ Incorporating citizen science into urban soil research fosters not only data collection but also the formation of “soil publics” - groups that recognize the importance of soil health and actively engage in addressing pollution and environmental justice issues.^22^ Projects such as *Our Soil* demonstrate how citizen science can generate a collective awareness of soil contamination, empowering communities to take local actions while transforming human-soil relations.^22^ By leveraging community participation, participatory science promotes inclusive inquiry and fosters environmental stewardship. With proper training, empowering participants improves data accuracy and nurtures a shared responsibility for environmental management.

The value of citizen science extends beyond data collection - it enhances decision-making processes by providing real-time, localized information^23^ and can help rebuild trust and ensure that soil management practices reflect the needs of those most affected by environmental challenges.^12^ In countries such as the USA, Australia, and the UK, citizen science programs have successfully mobilized volunteers to gather specific types of environmental data, delivering both snapshots and time series of changing conditions.^19^^,^^24^ Efforts such as the Harvard Law School Emmett Environmental Law and Policy Clinic have further empowered communities by developing manuals that guide community-based environmental data collection. These resources help participants not only contribute to scientific research but also advocate for environmental justice, making data accessible to communities for addressing local concerns.^25^ To ensure ethical engagement, reliable data collection, and meaningful outcomes, urban soil research initiatives can align with the European Citizen Science Association’s Ten Principles of Citizen Science,^26^ which outline best practices for participant involvement and project design. This approach fills data gaps, enriches knowledge, and enhances public engagement.^12^

Despite the success of participatory science in various fields, its adoption in soil science has been limited. Soils are less familiar to the general public in increasingly urbanized societies, in contrast to predominantly rural societies. Unlike birds or plants, soil is often less “attractive” for public engagement, and its hidden nature compared to the atmosphere or biosphere makes it less visible.^20^ Soil data collection can also involve labor-intensive fieldwork. Nevertheless, advances in technology make it feasible to involve the public in soil monitoring, encouraging a greater understanding of soils, fostering a commitment to conservation, and informing policymakers about soil conditions.^20^ While previous studies, such as Mason et al. (2023) and Head et al. (2020), have explored citizen science in agricultural and broader soil health contexts, its application to urban soils remains underdeveloped, despite the unique challenges posed by contamination, land use variability, and socio-economic disparities in cities.^27^^,^^28^

Thus, this perspective advocates for a broader adoption of participatory science as a pivotal tool in enhancing urban soil stewardship. By examining the unique benefits of participatory science, along with the challenges of effective integration, this work provides actionable strategies to increase participation and improve data quality. It presents a framework for fostering collaboration between scientists and urban communities, ensuring that participatory science initiatives are not only scientifically robust but also accessible and impactful. Ultimately, this perspective makes a case for scaling up participatory science efforts, highlighting its potential to empower communities, improve data collection, drive environmental education, inform policy decisions, and leverage digital technologies to promote sustainable urban living and advance environmental justice.

## Benefits, challenges, and strategic solutions in participatory science

### Community engagement and the benefits of participatory science

Historically, scientists have often developed and implemented solutions to environmental issues that diverge significantly from local community beliefs and practices. The inclusion of local community members in research teams and the active engagement in providing logistical support play a pivotal role in building trust between scientists and communities.^12^^,^^24^ This collaboration fosters cultural exchange, ensures the mutual recognition of scientific outcomes, and promotes the acceptance of research findings. By integrating community perspectives, participatory science initiatives can bridge the gap between scientific objectives and local needs, ensuring that solutions are culturally relevant and socially acceptable.

The Earthwatch Institute’s programs^24^ have identified four key spheres of activity that contribute to the success of citizen science initiatives by collectively enhancing natural and sociocultural capital:

1. Engaging people and key stakeholders (organizations and businesses become more sustainable; environmental leadership developed).
2. Informing global and local agendas (improved environmental policies, agendas, and management plans enacted and implemented).
3. Supporting important field research (scientific knowledge generated and shared).
4. Inspiring individual and community action (tangible local action).

These spheres are interdependent and often reinforce each other through feedback loops, enhancing the overall effectiveness of citizen science programs.^24^ This dynamic aligns with the Virtuous Cycle framework,^29^ where increased participation leads to greater community awareness, driving more impactful actions and generating lasting environmental and social benefits. When these spheres operate in harmony, they achieve lasting impacts by generating valuable goods and services (e.g., enhanced natural resources, ecosystem services, environmental data for planning, social cohesion, cultural preservation, and economic opportunities tied to sustainability and resource management) derived from both natural and anthropogenic environments.^24^ For example, scientific results can inform local actions and management plans, while management planning can guide scientific inquiries and community initiatives. Evaluating progress against the aims of these activities is crucial for demonstrating success to external assessors, including local partners, contributors, and funders.^24^ Citizen science projects measure success through participant engagement, sample collection, educational impact, behavioral change, and contributions to data-driven policy and community action.^23^^,^^30^^,^^31^^,^^32^

As the field of participatory science continues to grow and evolve, more robust strategies for evaluation, grounded in contextually appropriate and reliable assessment methods, are essential. These approaches will enable practitioners to design projects that enhance learning outcomes and accurately measure whether participants are achieving specific educational and engagement goals. Such evaluations may include assessing the participants’ comprehension of scientific processes, their ability to interpret data, and the extent to which the project meets its intended research and social impact objectives.^33^ Bonney et al. (2016) proposed the following learning outcomes as feasible and measurable outcomes of citizen science projects based on interviews and surveys with participants.

1. Curiosity in science and nature
2. Self-efficacy for science and ecological action
3. Enthusiasm for science and environmental action
4. Skills of science investigation
5. Data interpretation skills
6. Understanding of the nature of science.

### Challenges in data quality and scientific integration

Yet, one of the primary challenges in participatory science is effectively integrating observations from citizens with those from professional scientists. According to Rossiter et al. (2015), citizen science observations related to soil include a range of data types, including surficial (e.g., photos, surface features, landscape description) and subsurface descriptions (e.g., pits and profiles), field measurements (e.g., soil texture and pH levels using kits), instant sensor results, and basic laboratory analyses.^20^ Volunteers may also provide field measurements, such as soil texture and pH levels using kits, alongside sensor readings and basic laboratory analyses.

Still, data collected by the public are often more general, readily available, but typically less precise than data obtained by trained surveyors using sophisticated instruments and advanced laboratory techniques. Soil sampling, in particular, presents unique challenges that make it inherently more labor-intensive than other types of environmental sampling, such as plant surveys. Collecting soil samples at depth requires significant physical effort, specialized tools such as augers or coring devices, and a solid understanding of proper sampling techniques. These factors contribute to the lower precision in volunteer-contributed data.

Moreover, these challenges are compounded when volunteers are expected to adhere to complex data collection protocols, which can lead to inconsistent sampling efforts and participant biases. For example, biases linked to socioeconomic factors can significantly influence the spatial distribution and quality of community-contributed data. Research has demonstrated that areas with higher socioeconomic status are often better sampled than lower-income areas, resulting in spatial biases that affect the representativeness of the data.^34^ Simplifying data collection protocols and addressing the physical demands of soil sampling can improve participation rates, reduce biases, and ultimately enhance the reliability of citizen-supplied data.^35^^,^^36^ It is also important to include cultural competency education as part of volunteer training to ensure participants understand and effectively engage with the communities they are working in.

Rossiter et al. (2015) raise two critical questions about the use of citizen science data^20^: “(1) How can the credibility of citizen-supplied observations be assessed? (2) How can imprecise observations be combined with professionally provided observations?”

### Strengthening data reliability through technology and mapping

Common issues affecting the reliability of volunteer-supplied data include locational inaccuracies, accuracy of descriptions or measurements, and inconsistencies between participants. These problems are prevalent across many participatory science projects. For example, the Cornell Lab of Ornithology’s eBird project uses a combination of automated data filters and expert reviews to assess bird species observations.^37^ These filters are designed to flag anomalies, such as reports of species outside their typical habitats. A similar system could be applied to soil surveys, where an alert could be triggered if an unusual soil type, such as Histosols (organically rich soils), is reported in an environment where it is not expected.^20^ In such cases, the flagged data could be reviewed by experts, and citizens could be asked to provide photographic evidence to verify their observations.^20^

As web mapping applications continue to evolve, greater emphasis must be placed on their usability, user experience, task requirements, data accuracy, and suitability for the intended purpose and audience they aim to support.^38^ For participatory scientists to effectively use interactive web mapping applications, they must possess a certain level of spatial literacy. This includes the ability to understand spatial relations, visualize locations, and make informed decisions using geographic information. Additionally, while default GPS settings are often sufficient for basic geolocation tasks, understanding concepts such as datums, projections, map units, and resolution becomes important when integrating data from multiple sources or working on projects requiring higher spatial precision.

One way to assess the accuracy of volunteer-provided data is by comparing new observations with existing soil-landscape models and predictions derived from current maps while accounting for associated uncertainties. For example, if a new observation falls within the confidence interval of a kriging prediction, it could be considered reliable.^20^

To further improve data reliability and utility, it is essential that soil samples collected by communities be georeferenced and added to a shared mapping system. This common map would allow participants and researchers to locate samples spatially across neighborhoods and cities, helping to identify spatial and temporal patterns. A shared, open-access georeferenced map would serve several purposes: (1) enable community groups to compare their neighborhood or city to others, (2) contribute to a georeferenced database that could help researchers identify larger patterns, and (3) allow for tracking anomalies or outliers in soil types or contamination levels. A good example of such an approach is the CoCoRaHS precipitation network,^39^ where citizen-reported rain gauge data are incorporated into NOAA’s precipitation maps, improving trend analysis and community comparisons. A similar strategy could greatly improve the integration of soil data from volunteers and professionals alike. Citizen science networks such as the Global Urban Soil Ecology and Education Network (GLUSEEN)^40^ have demonstrated the potential of collaborative data collection to enhance the understanding of urban soil systems across multiple cities and biomes.^21^

Participatory science platforms play an essential role in simplifying data collection and contribution, ensuring that participants’ lack of fundamental knowledge does not impede the project’s success. These platforms are critical in advancing web mapping projects and will be pivotal for future geographic information science (GIScience) research.^41^ To achieve this, websites and tools must be user-friendly, offer positive experiences for a diverse range of users, support multiple tasks, and maintain high data quality.^41^ By implementing strategic quality control measures, leveraging digital tools, and ensuring inclusive participation, participatory science can drive evidence-based policies, improve urban soil health, and create long-term environmental and social benefits.

## Empowering communities through participatory science programs involved with urban soils

Engaging communities in participatory science initiatives requires both effective communication and sustained support. Case studies from various regions illustrate how successful programs have fostered strong community relationships while generating valuable environmental data.

### Large-scale participatory science initiatives

The *VegeSafe* and *DustSafe* programs are large-scale citizen science initiatives that have collected 26,500 soil and dust samples from 7,200 homes across Australia, expanding to 35 countries globally.^31^ Participants were recruited through emails, social media, word of mouth, media coverage, and community events, with word of mouth (31%) and the program website (23%) being the most common sources. A survey of 522 participants showed that 93% found the information useful, 76% felt more involved in science, and 40% took actions to mitigate exposure, such as using raised garden beds and improving indoor hygiene. The programs, designed to be accessible with free or low-cost testing, attracted primarily educated homeowners (79%), with 65% female participants. Feedback led to improvements in data presentation, including visual aids and online mapping tools. The initiatives have influenced public health policies, supported research through global partnerships, and increased community engagement with environmental health, exemplifying successful citizen science.^42^^,^^43^

The GLOBE Program is an excellent example of a successful global citizen science initiative, with participants from 112 countries contributing over 100 million measurements since its inception in 1995.^44^ The program includes 58,000 teachers and 1.5 million students, with participating students often performing better in state science exams and competitions. For instance, in California, students petitioned their city council to preserve a wooded area for educational purposes, preventing its sale to real estate developers.^44^ The *GLOBE* website (https://www.globe.gov/web/soil) offers 16 pedosphere protocols, enabling students and educators to collect critical soil information that contributes to global soil health research and awareness.

The *MapMyEnvironment* program – web portal offers free environmental testing for heavy metals in soil, dust, and paint, providing tailored mitigation recommendations, educational resources, and live maps for public access to heavy metal data globally. Its database from the U.S. and other countries includes over 23,000 soil samples and 2,900 indoor dust samples, providing property-specific environmental data contributed by volunteers—data that would be challenging to collect through traditional research methods.^45^

### Community-led projects in urban soil contamination research

*Project Harvest* was a citizen science initiative that engaged 120 diverse participants and 7 promotoras across Arizona to monitor contaminants in harvested rainwater, soil, and plants, aiming to increase environmental health literacy and inform local action.^32^ Over 50% of participants reported increased confidence in performing scientific tasks, with 38% joining due to an interest in gardening and rainwater harvesting and 24% seeking information on environmental contaminants. While many participants felt empowered, some—particularly in rural areas—faced challenges with technology and time constraints, contributing to 23 resignations linked to these barriers.^32^

In Lafayette, Louisiana, over 100 residents submitted 763 soil samples as part of a community project, receiving remediation advice and demonstrating the value of ongoing communication for maintaining community engagement and trust.^46^ These programs underscore the long-term potential of citizen science to inform urban planning and environmental policy, empowering communities to manage their health risks more effectively. In Peru, a community-led project involved parents in mining towns sampling soil for lead contamination in children’s play areas, uncovering new hotspots, and empowering families to take action.^47^

### Community projects exploring life belowground

Incorporating participatory science into urban soil research has also proven effective in biodiversity data collection, as shown by *Earthworm Watch*, which provided valuable insights into earthworm distribution in urban environments, despite challenges posed by varying participant skill levels.^48^ *Jardibiodiv*, a citizen science project launched in 2017, successfully engaged approximately 50 adults and at least 20 school classes predominantly implemented in northeastern France.^49^ The project revealed significant urban soil biodiversity, identifying 5,050 collembolans and 9,107 macroinvertebrates over two years, with ants, spiders, and woodlice dominating in gardens and notable diversity variations across land-use types.^49^

The *Tea Bag Index* project in the UK enlisted over 500 participants to study decomposition rates in domestic gardens, demonstrating how urban soils contribute to carbon cycling and climate change mitigation.^50^ Key findings revealed that soil amendments significantly boosted soil carbon and nitrogen levels, with individual garden practices having a greater impact on carbon storage than geographic location – insights directly applicable to urban sustainability.^50^ This method has also been widely adopted in participatory science projects around the world, including in schools, making it an accessible and scalable tool for public engagement in soil science. Overall, these studies highlight the critical role of participatory science in uncovering urban soil biodiversity patterns and related processes to inform sustainable soil management strategies.

### Participatory science shaping urban policies and food systems

Participatory science has not only contributed to scientific data collection but has also influenced urban policies and regulations. Grassroots movements focusing on environmental projects and community gardens have shaped land use decisions. For instance, Boston’s *Grassroots* and *Open Space Development Project*^51^ funded community gardens in low-income neighborhoods, transforming urban spaces into areas for food production and green infrastructure. Similarly, grassroots anti-deforestation efforts in cities such as New Delhi have prevented large-scale tree removals, directly impacting urban forestry policies. The Center for Neighborhood Technology (CNT)^51^^,^^52^ enables communities with data tools to advocate for better water management policies, highlighting the role of community engagement in driving urban environmental improvements.

Urban food production also serves as a vital link between communities and soil systems, particularly in addressing urban soil health. A lack of education on soil health, however, exacerbates the risks of soil contamination in urban areas. Contaminants such as lead, arsenic, and cadmium pose significant dangers to public health,^53^^,^^54^^,^^55^ but these risks can be mitigated by implementing sustainable soil management practices such as regular soil testing and remediation.^10^^,^^56^^,^^57^^,^^58^ For urban communities involved in agriculture, access to soil health information—including historical land use data, contamination levels, and testing protocols—is essential.^3^^,^^12^ When paired with remediation guidance and exposure reduction strategies, this knowledge forms the foundation for ensuring safe and sustainable urban food production.^3^ The *Edible Schoolyard Project*^59^ in Berkeley, CA, educates students about food production, soil ecosystems, and nutrition through gardening. *City Slicker Farms*^60^ in West Oakland and the *Detroit Black Community Food Security Network*^61^ empower local communities by establishing urban farms, providing education on sustainable agriculture, and enhancing access to fresh, affordable produce, thereby fostering food security and community engagement.

Through such initiatives, organizations aim to enhance public understanding of sustainable farming, gardening, and soil health management, fostering community engagement and knowledge-sharing. The inclusion of local knowledge from the outset of these programs ensures that environmental assessments are contextually and culturally relevant, gaining stronger local support and leading to more comprehensive solutions.^24^ While not explicitly used in urban soil participatory-science projects, the principles of Asset-Based Community Development (ABCD)—which focus on leveraging community strengths, relationships, and local knowledge rather than addressing only deficits—could further enhance their impact by expanding participation and fostering community-driven solutions.^62^ As Schwarz et al. (2022) emphasize, early, sustained, and inclusive community engagement is key to achieving true co-development and co-empowerment. This approach enables the integration of local knowledge from those actively managing urban soil systems, which enhances both the relevance and effectiveness of environmental assessments. Engaging communities early—during the research question or priority-setting stage—communicates a commitment to community-centered approaches, though it may also introduce challenges if community-driven priorities diverge from those set by funding agencies.^12^

Participants in citizen science initiatives gain practical knowledge about soil resources by conducting hands-on soil testing and contributing to data collection efforts.^20^ Soil survey organizations can find flaws or needs in their own professional projects and maps by comparing volunteer-provided information to their own products and identify the areas that require updating. This collaborative approach enables participants to influence the data generation priorities of government, creating an enhanced map that targets areas in greatest need of attention.^20^

## Strengthening urban soil stewardship through creative and educational initiatives

Educational resources play a pivotal role in fostering community involvement in urban soil stewardship.^8^^,^^63^ A global study of soil science education in 43 countries found significant disparities in high school textbook content, indicating better soil science education in Mongolia, Brazil, Turkey, Tunisia, Niger, Uganda, Uzbekistan, and Italy.^64^ Soil genesis and soil profile were the most covered topics, while world soils and agricultural usefulness received the least attention with no discussion of urban soils or city environments.^64^ This gap highlights the need for targeted educational initiatives that address urban soil challenges and engage communities in soil stewardship. Organizations such as the U.S. Department of Agriculture (USDA) educate urban residents on soil health through Natural Resources Conservation Service (NRCS) lesson plans,^65^ urban soil surveys,^66^ and support for urban agriculture,^67^ providing resources and technical assistance for sustainable soil management. The Soil Science Society of America provides curricula that focus on the impacts of human activity on soil systems, as well as strategies for protecting and improving urban soils.^68^

In New York City, both the Urban Soils Institute^69^ and Brooklyn College’s Urban Soils Lab^70^ offer initiatives to educate communities on soil health, particularly through soil testing for heavy metal contamination and workshops in collaboration with non-profit organizations and government agencies. Using portable X-ray Fluorescence (XRF) analyzers, these organizations provide quick, cost-effective screenings and remediation guidance via lab services and community events. In Indiana, the Urban Soil Health Program^71^ focuses on improving soil health for urban and small-scale agricultural lands by offering technical assistance, workshops, and site visits to urban growers. The program encourages practices that minimize soil disturbance and promote biodiversity, both of which are crucial for maintaining healthy urban soils. In California, TreePeople^72^ engages communities in urban soil health by combining education with volunteer-driven projects focused on urban forestry, watershed management, and soil health.^73^ Their programs improve soil quality and enhance urban landscapes, addressing urban soil degradation through sustainable land management practices.

Digital platforms, such as blogs, provide scientists with a flexible space to communicate their research, share techniques, and engage with the public. Blogging offers real-time interaction, making it easier to explain the complexities of scientific topics to a broader audience.^74^ For example, the TreePeople blog^75^ features insights on urban soil health and its role in sustainable cities, while the “Digging Deep” blog^76^ by The Urban Soil Doctor provides practical soil insights tailored to landscape professionals and gardening enthusiasts. However, such blogs remain relatively rare. Blogs can amplify the voices of marginalized communities, particularly in discussions of environmental justice, where community engagement and local knowledge are essential (Table 1).

**Table 1:** Summary of strategies, resources, implementations, and case examples in urban soil science, highlighting key attributes for successful participatory science initiatives, including community engagement, accessibility, technological innovation, education, and collaboration

Strategy and resources	Implementation	Case examples	Community engagement	Public accessibility	Technological innovation	Educational value	Collaborative efforts
Workshop series	Conduct workshops on soil properties, health indicators, and their role in urban environments. Public participation in soil sampling and data collection under scientific guidance.	https://www.urbansoilhealth.org/events-1 https://urbansoils.org/new-events https://www.atsdr.cdc.gov/soilshop/index.html	✔	✔		✔	✔
Expert seminars	Host seminars led by soil scientists to discuss the latest urban soil research and management practices.	https://halfmoonseminars.org/product/webinars/understanding-and-working-with-urban-soils/ https://bwsr.state.mn.us/tech-talk-intro-urban-soil-health-conservation-planning-and-management https://connect.extension.org/g/nuel/blog/urban-soils-webinar	✔	✔		✔	✔
Exhibitions and installations	Host exhibitions and public installations that combine science and art to visually communicate the importance of urban soil health. These events should be held at public venues and designed to attract and educate a broad audience.	“Dig It! The Secrets of Soil” exhibit from the Smithsonian’s National Museum of Natural History, USA “Soil and Forest Biodiversity” collection, Spain “Urban Soils Room at Swale House” exhibition, USA “The Home Soil” exhibition, Australia https://wsm.isric.org/WSRC.html	✔	✔		✔	✔
App integration	Collaborate with mobile app developers to include educational features such as interactive guides and gamified learning for soil data collection, as well as utilize soil apps for displaying testing results and facilitating data exploration.	Apps https://soilexplorer.net/ https://casoilresource.lawr.ucdavis.edu/gmap/ https://microbiometer.com/our-test/ https://www.isric.org/news/sqapp-launch https://www.spotteron.net/blog-and-news/introducing-the-apps-the-tea-bag-index			✔	✔	
Social media engagement	Regular updates, live sessions, and interactive content. Share project findings and educational materials through engaging posts, videos, and infographics. Launch digital campaigns via vlogs to raise awareness about urban soil health. Utilize hashtags and collaborate with influencers to expand reach and visibility.	Platforms Instagram, Facebook, TikTok, and Twitter Blogs https://dynamicecology.wordpress.com/ https://sustainable-secure-food-blog.com/editorial-page/ https://soilhealthbenchmarks.eu/benchmarks-blog/ https://www.instagram.com/p/Cq5r_QPtDck/	✔	✔	✔	✔	
Online forums, data platforms and communities	Participate in or create online forums and groups to foster discussions, share soil science experiences, and build a community of practice based on peer-to-peer learning.	Platforms such as Facebook and Reddit https://www.lurzain.eus/home https://teacomposition.sydney.edu.au/map/ http://www.gluseen.org/ https://elgg.org/	✔	✔	✔		
School programs	Partner with schools to integrate soil science into curricula, engaging students through projects.	https://agupubs.onlinelibrary.wiley.com/doi/full/10.1029/2021GH000498 https://edibleschoolyard.org/	✔			✔	✔
University collaborations	Work with universities to involve students in participatory science projects as part of their coursework or research.	https://australiancotton.com.au/news/soil-your-what-industrys-unique-way-to-test-soil-health https://urbanhealth.indianapolis.iu.edu/projects/soil-testing-for-lead	✔			✔	✔
Garden clubs and community gardens	Provide resources and information to local gardening clubs and community gardens, encouraging soil testing and data sharing among members.	https://agriculture.ny.gov/news/state-department-agriculture-announces-2024-new-york-state-community-gardens-soil-testing https://errin.eu/calls/living-labs-urban-areas-healthy-soils	✔	✔		✔	✔
Accessible literature and video content	Develop easy-to-understand materials such as videos, infographics and manuals to educate the public on soil testing and management.	Resources: USDA NRCS https://www.nrcs.usda.gov/getting-assistance/other-topics/urban-agriculture Urban Soil Primer https://www.nrcs.usda.gov/sites/default/files/2023-01/Urban-Soil-Primer-Homeowners-and-Renters.pdf Urban Soil Management for Climate Resilience https://www.treepeople.org/wp-content/uploads/2023/03/urban-soil-management-for-climate-resilience-report.pdf The Urban Soil Guide: A Field and Lab Manual by Anna Paltseva https://content.ces.ncsu.edu/minimizing-risks-of-soil-contaminants-in-urban-gardens https://blogs.cornell.edu/healthysoils/ https://treepeople.org/healthy-soils-for-healthy-communities-initiative/ https://nrcs.maps.arcgis.com/apps/MapJournal/index.html?appid=b38d8cc57afa4a2f88bc5dd65791c7c2 YouTube channels: https://www.youtube.com/@epicgardening https://www.youtube.com/@TheUrbanFarmingGuys https://www.youtube.com/@TreePeople www.youtube.com/@UrbanSoils https://www.youtube.com/@annapaltseva		✔	✔	✔	

**Figure 1:**
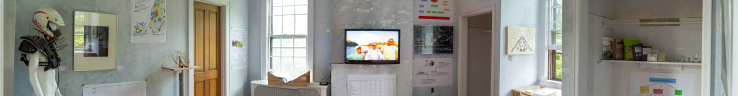
Exhibition art and science of soil and gardens by Donald Daedalus and Anna Paltseva at Swale House, Governor’s Island, NYC in 2019 Photo credit: Don Hải Phú Daedalus.

Art-based initiatives also raise awareness of urban soils and soil contamination in particular. Margaret Boozer’s *Correlation Boxes*,^77^ a collaboration with a soil scientist from the USDA, involved collecting soil samples from all five boroughs of New York City. This project was first exhibited in 2012 at the Museum of Arts and Design in NYC, using plexiglass boxes to visually map the diversity and significance of urban soils. Boozer expanded the project by creating the permanent *Urban Soils Room* on Governors Island, NYC, to educate the public on soil health, food justice, and environmental sustainability. In the meantime, the *Illinois River Project*^78^ by Donald Daedalus connects ecological restoration with social justice by proposing to employ individuals with criminal histories to process invasive species into compost, used to remediate lead-contaminated soils in urban areas. An exhibition curated by Daedalus and the author in New York City (Figure 1) combined science and community-driven solutions to address urban environmental issues, highlighting how soil remediation techniques, contamination data, and the ecological value of composting can improve soil health. Similarly, creative initiatives such as *Fundred’s Hundred Dollar Bill Project*^79^ led by Mel Chin advocate for investments in soil remediation, particularly targeting lead-contaminated soils, using art as a tool for raising awareness and driving action. By integrating education, digital platforms, and creative initiatives, urban soil stewardship can be strengthened, ensuring that science, policy, and community engagement work together to promote healthier and more sustainable urban environments.

## Key strategies and tools for enhancing public engagement in urban soil science

Effective public education on soil science is essential for fostering environmental stewardship and ensuring the success of citizen science initiatives.^80^ The primary goal is to increase soil literacy among urban residents, encouraging them to actively participate in soil data collection and conservation. To achieve this, adopting an intentional approach to science communication is crucial. As Kantar et al. (2023) argue, intentional communication strategies—tailored to different audience needs—can help bridge gaps between complex scientific concepts and public understanding, which is especially important in engaging diverse communities in urban soil science.^81^ Post-sampling engagement can help maintain community interest and investment in the project.^36^ By involving participants in the interpretation of results, discussions on potential remediation strategies, co-developing action plans, hosting community data-sharing events, citizen science not only improves data accuracy but also sustains engagement.^31^^,^^32^^,^^36^ To reach and engage a broad audience, a combination of traditional educational approaches and innovative digital tools is necessary. Similar to how soil museums contribute to public understanding of soils by making scientific knowledge more accessible,^82^ participatory science initiatives in urban soil research can engage communities through interactive, accessible platforms that foster awareness and participation. Later in discussion are some strategies that can be implemented to facilitate data collection and promote soil stewardship (Table 1).

To ensure the successful implementation of these strategies, several critical elements of an urban soil participatory science project must be considered, all of which enhance the common success attributes highlighted in Table 1:

1. Resource allocation: Adequate financial and material resources are essential to support educational initiatives, from workshop materials and video creation to app development and event coordination. Proper resource allocation allows for sustainable community engagement and the continued development of innovative technologies. Examples of funding sources are the Knight Foundation, Toyota, National Fish and Wildlife Foundation, The Conservation Fund, Earthwatch Institute, The Bush Foundation, ASTC’s Community Science Initiative, USDA Forest Service Citizen Science Competitive Funding Program, NASA’s Citizen Science for Earth Systems Program, and so forth.
2. Collaborative efforts: Collaboration with local governments, NGOs, schools, universities, and community leaders is vital to expanding the reach of these strategies and gaining public support. These partnerships foster more extensive public engagement and help integrate participatory science into local environmental policies and education.
3. Feedback mechanisms: Establishing channels for participant feedback allows for continuous improvement and adaptation of initiatives. Feedback not only refines future efforts but also helps maintain long-term community engagement by fostering a sense of shared ownership and responsibility in the project.

By integrating these elements into urban soil research efforts, participatory science can become an embedded part of community life. This approach will cultivate environments where individuals from all backgrounds can actively contribute to a deeper understanding of urban soil health and take part in meaningful environmental action. A useful model for sustaining urban soil initiatives is the Virtuous Cycle framework,^29^ which shows how increased community involvement enhances awareness, drives more effective action, and improves outcomes. As communities witness tangible benefits from their participation, their engagement deepens, creating a reinforcing loop of education, action, and positive environmental impact.^29^ Furthermore, the emphasis on resource allocation, collaboration, and feedback will help sustain community-driven research and amplify its impact in promoting sustainable urban development.

### Community building and experience sharing with digital technologies and social media

Upcoming technologies are set to revolutionize participatory science by engaging previously uninvolved groups.^30^^,^^83^ Younger, diverse citizens will drive this change through networked and open science and gaming applications, altering how scientific discoveries are made. Global science programs, mobile/web-based activities, and large-scale data visualizations are increasingly attracting broader audiences. Participatory-science video games may soon become part of after-school programs, and affordable tablets are enabling remote communities to contribute data, fostering a population of always-connected, data-aware individuals. This integration blurs the line between daily life and scientific participation.^83^^,^^84^

Advancements in citizen science mobile apps have further created a “digital agora” – a collaborative space where public engagement extends beyond data collection to shaping policy and urban development.^84^ These apps enhance geospatial tools and real-time tracking of environmental risks, enabling participants to map contamination hotspots with greater precision.^84^ The ECHO project,^85^ co-funded by the European Union and UK Research and Innovation, exemplifies how citizen science can drive soil health awareness and promote behavioral change across the EU. ECHO enables citizens to take an active role in soil science, contributing to data collection at 16,500 sites across Europe and Scotland. The data, accessible through ECHOREPO, will be linked to the EU Soil Observatory and used by scientists, policymakers, and the public to inform critical decisions about soil health and conservation efforts.

The integration of big data—characterized by its high volume, variety, and velocity—into citizen science projects is transforming urban sustainability efforts.^30^ By broadening the scope of data collection, community-sourced data are providing critical insights for long-term urban planning and policy. Initiatives such as the New York City Street Tree Map exemplify how citizen science can contribute not only to immediate environmental monitoring but also to strategic urban decision-making through extensive community engagement.^30^

Social media and digital platforms are powerful tools for enhancing educational outreach and raising awareness about urban soil health. By leveraging these platforms strategically, participatory science initiatives can engage broader audiences and build informed communities committed to soil stewardship (Table 2). Additionally, digital platforms facilitate granular data collection, capturing demographic and behavioral insights that help refine engagement strategies. For instance, the B.E. Smart project in Rome used web-based platforms to gather energy consumption data, a model that can be adapted for soil health initiatives to better target citizen participation.^30^^,^^84^

**Table 2:** Benefits of social media in enhancing urban soil health projects

Benefits of social media	Description
Educational outreach and awareness	Platforms such as Instagram, X, and Facebook can disseminate information through infographics, short videos, and posts explaining the importance of urban soil health, soil science basics, and how volunteers can participate in data collection.
Real-time updates and engagement	Social media can provide real-time updates on project progress, keeping the community informed and engaged. Live Q&A sessions or live-streaming events allow for direct interaction with the audience.
Community building and experience sharing	Facebook Groups or Reddit forums enable participants to share their soil sampling experiences, results, and observations, fostering peer-to-peer learning and ongoing participation.
Data visualization and sharing results	Instagram and Pinterest can be used to share visual content such as maps, before-and-after images, and data visualizations of soil remediation projects, illustrating project impact. Web Soil Survey can be used for soil maps and reports to understand the background.
Collaborative learning	YouTube channels can host tutorials, educational videos on soil science, and interviews with experts, providing an accessible platform for learning and broadening the project’s audience.
Leveraging influencers and partnerships	Collaborating with influencers or organizations with a strong social media presence can amplify project reach, encouraging more people to participate and share educational content.
Hashtag campaigns for wider reach	Unique hashtags can increase project visibility, making it easier to track discussions, data collection efforts, and shared experiences.

Leveraging mobile apps not only allows volunteers to participate actively in data collection but also fosters continuous engagement in a “digital agora,” encouraging dialogue between participants and policymakers about urban environmental issues. By integrating both social media platforms and innovative mobile apps, participatory science projects can achieve a higher level of engagement, with technology bridging the gap between scientific communities and the general public, fostering ongoing environmental stewardship.

## Framework for urban soil and participatory science collaboration and engagement

**Figure 2:**
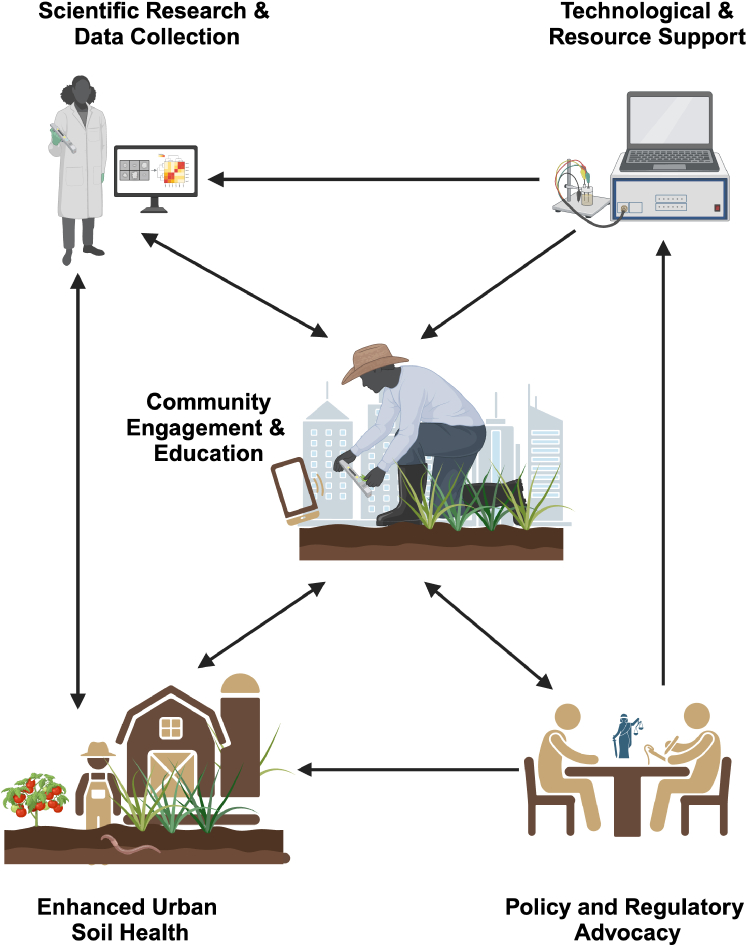
Framework for community-driven urban soil research and policy action Created in BioRender. Paltseva, A. (2025) https://BioRender.com/t68u778.

Participatory science enhances urban soil research by improving data collection, engaging communities, and shaping policy. Community-driven initiatives generate critical data on soil health and environmental risks, informing both local actions and broader policy reform. This collaborative approach integrates scientific research, community involvement, policy advocacy, and technological innovation, forming a framework for advancing urban soil research and improving environmental outcomes. Figure 2 presents a framework for community-driven urban soil research and policy action, illustrating the interdependent roles of four key components:

1. Scientific Research and Data Collection – Establishes standardized methods and datasets that inform urban soil health assessments and sustainable land management strategies.
2. Technological and Resource Support – Provides advanced soil testing tools, digital platforms, and adequate funding to enhance data accessibility and research efficiency.
3. Community Engagement and Education – Promotes scientific literacy, assesses local soil concerns, and fosters active public participation in data collection and stewardship.
4. Policy and Regulatory Advocacy – Bridges research and governance by informing policymakers and promoting science-based regulations for equitable urban soil management.

**Figure 3:**
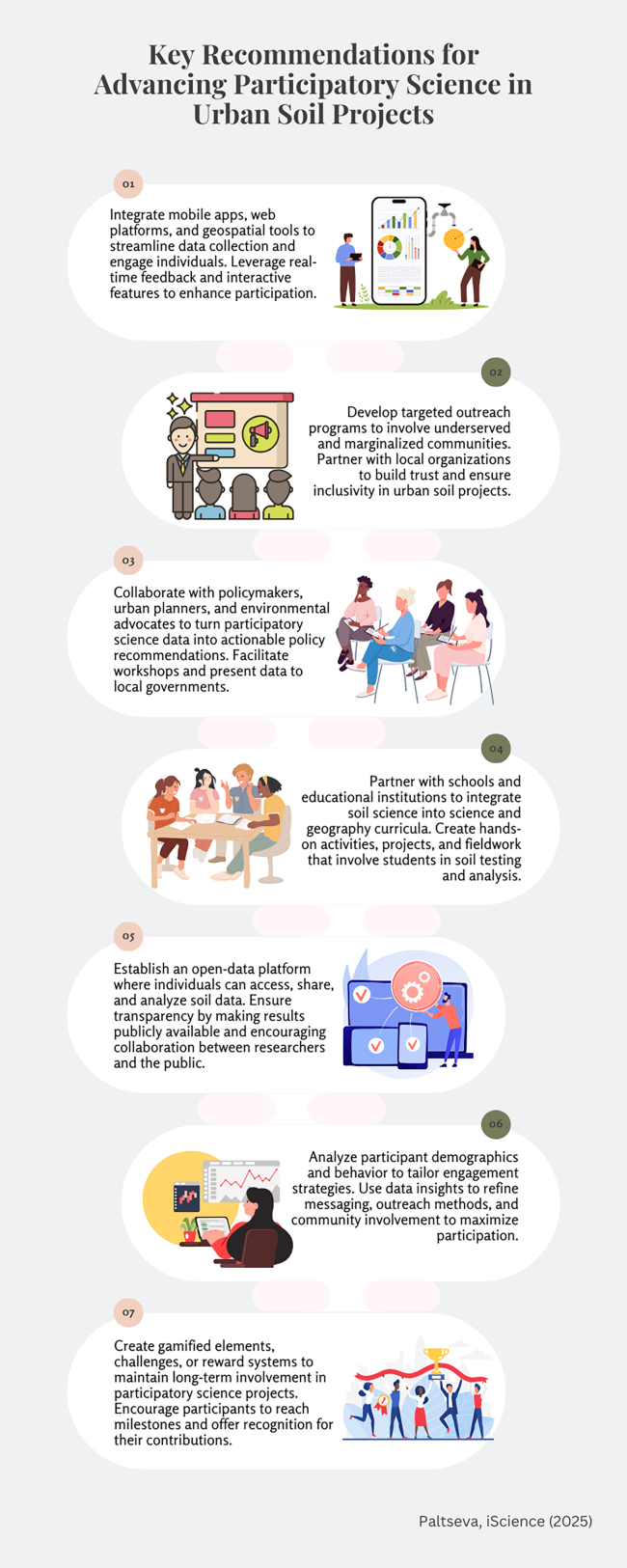
Key strategies to enhance participatory science engagement in urban soil projects, focusing on participation, inclusivity, and collaboration

Implementing the framework and its recommendations (Figure 3) can significantly advance participatory science in urban soil research. A collaborative, multi-faceted strategy is essential to addressing challenges and leveraging opportunities, ensuring participatory science remains a powerful tool for urban soil stewardship and sustainability.

## Limitations of study

This participatory science framework for urban soil research presents several practical limitations. Data accuracy can vary based on participants’ experience, consistency in following sampling protocols, and levels of technological proficiency. Reliance on digital tools assumes participants have adequate access to internet services and sufficient technical literacy, which might not be uniformly available across diverse urban communities. Socioeconomic factors could lead to uneven participation and spatial biases, potentially affecting the representativeness of the collected data. Additionally, the physically demanding nature of subsurface soil sampling may hinder long-term community engagement, reducing the reliability and completeness of data collection.

## Conclusion

Urban soils hold immense potential for biodiversity, pollution mitigation, and urban agriculture, yet they remain underutilized due to limited public awareness, data gaps, and inconsistent management. Participatory science offers an innovative solution by enlisting local communities in soil stewardship, bridging critical data shortfalls, and fostering a shared sense of environmental responsibility. By involving non-scientists in real-time data collection, participatory science not only strengthens scientific knowledge but also cultivates public trust and engagement.

Nevertheless, sustaining data reliability and participation demands simple, well-validated protocols and supportive technologies, such as mobile apps and open-data repositories. These tools make soil monitoring more interactive, inform policy decisions, and facilitate targeted remediation actions. Universities and other institutions can further boost these initiatives by broadening their definition of scholarly impact—valuing outreach and public collaboration alongside academic publishing. Adopting frameworks such as the “Third Mission”^86^ underlines the societal value of research, encouraging deeper partnerships between scientists, policymakers, and communities.

To move from concept to action, researchers can replicate or adapt tested participant-science protocols in new urban contexts, while local governments should integrate community-generated data into planning and environmental regulations. Nonprofit organizations and community groups are likewise encouraged to establish partnerships with scientists, securing resources and political support for soil-health initiatives. Ultimately, a holistic approach—integrating technical innovation, stakeholder engagement, policy advocacy, and inclusive funding—ensures that participatory science continues to drive meaningful progress in urban soil management. Through iterative feedback, transparent practices, and creative outreach, participatory science can remain a catalyst for environmental justice, public health, and sustainable urban development for generations to come.

## Acknowledgments

I would like to express my gratitude to Dr. Richard Pouyat, Dr. Michalis Charilaou, and Maxine Levin for their invaluable feedback and insightful suggestions, which greatly contributed to the improvement of this article.

## Author contributions

Conceptualization; methodology; visualization; writing—original draft preparation; and writing—review and editing, A.P.

## Declaration of interests

The author declares no competing interests.

## Declaration of generative AI and AI-assisted technologies in the writing process

During the preparation of this work, the author used ChatGPT to improve the readability and language of the article. After using this service, the author reviewed and edited the content as needed and took full responsibility for the content of the publication.
